# Glycometabolic burden and myocardial involvement: a relational two-axis hypothesis

**DOI:** 10.3389/fendo.2026.1989060

**Published:** 2026-09-30

**Authors:** Yebin Xia, Wenjie Zhang, Jinjian Guo

**Affiliations:** Department of Cardiology, The Second Affiliated Hospital of Fujian University of Traditional Chinese Medicine, Fuzhou, Fujian, China

**Keywords:** cardiac biomarkers, diabetic cardiomyopathy, diabetic myocardial disorder, heart failure, prediabetes

## 1 Introduction

Diabetic cardiomyopathy traditionally described myocardial abnormalities in diabetes without major alternative explanations, such as coronary artery disease or hypertension. The 2024 Heart Failure Association of the European Society of Cardiology consensus broadened this concept to diabetic myocardial disorder, recognizing that diabetes rarely acts alone. Obesity, hypertension, chronic kidney disease, and coronary artery disease often coexist and may jointly contribute to myocardial impairment (1). Community-based longitudinal research also linked persistent or worsening diastolic dysfunction to subsequent heart failure, supporting attention to myocardial abnormalities before clinical presentation (2).

Diabetes is defined by clinically useful glycemic thresholds, but these thresholds need not correspond to discrete myocardial transitions. The ACE 1950 study identified associations between glycated hemoglobin (HbA1c) and subclinical cardiac abnormalities, including below the diagnostic threshold for diabetes (3). We propose a relational two-axis hypothesis that treats paired metabolic–myocardial history as a prespecified target of longitudinal inference. It asks whether preceding paired trajectories retain reproducible information about subsequent myocardial progression beyond contemporaneous measurements and established clinical factors.

## 2 Clinical evidence and its limits

Longitudinal evidence supports a connection between metabolic exposure and myocardial disease. In the Atherosclerosis Risk in Communities (ARIC) study, prediabetes and diabetes were associated with incident high-sensitivity cardiac troponin T elevation over six years (4). The Coronary Artery Risk Development in Young Adults (CARDIA) study related long-term glycemic abnormalities and insulin resistance trajectories to midlife ventricular structure and function (5). These findings support exposure-related myocardial involvement rather than biological independence.

Within conventional glycemic categories, however, cardiac phenotype and prognosis remain heterogeneous. A pooled analysis stratified heart failure risk among adults with prediabetes or diabetes using a multimarker profile (6). An analysis of the Systolic Blood Pressure Intervention Trial found that prediabetes combined with elevated or rising cardiac biomarkers was associated with higher incident heart failure risk among adults with hypertension without diabetes (7). The Swedish Cardiopulmonary Bioimage Study found similar prevalence of abnormal diastolic measures in prediabetes and diabetes, with accompanying associations involving other cardiometabolic risk factors (8). These studies demonstrate substantial heterogeneity within conventional glycemic categories. Longitudinal observations also suggest variable temporal coupling. Pofi et al. observed worsening CMR-derived strain over five years despite unchanged reported metabolic parameters in type 2 diabetes (9). Ringle et al. reported modest worsening of echocardiographic GLS over six years in asymptomatic type 1 diabetes without other cardiovascular risk factors, while metabolic status did not correlate with GLS (10). Bogdanović et al. found persistent strain impairment three months after euglycemia was restored (11), whereas Leung et al. observed improved GLS with improved glycemic control over 12 months (12). These findings motivate direct testing of metabolic–myocardial trajectories rather than establish independent biological processes.

## 3 A two-axis hypothesis

We conceptualize glycometabolic burden and myocardial involvement as separately characterized, continuous dimensions. Glycometabolic burden encompasses the severity and duration of dysglycemia and insulin resistance, rather than diagnostic category alone. Prediabetes and diabetes remain useful clinical anchors, but exposure within each category is heterogeneous. Myocardial involvement comprises objectively detectable injury, stress, and structural or functional impairment. These manifestations are overlapping indicators, not a fixed sequential cascade. Neither axis constitutes a disease stage. Their joint position describes the paired metabolic–myocardial phenotype.

The hypothesis is that these biologically connected dimensions need not maintain a fixed relationship across individuals or over time. Separate characterization refers to measurement, not statistical or biological independence. The terms myocardial-predominant and metabolic-predominant are illustrative relational descriptors, not validated phenotypes, clinical categories, or mechanistic endotypes (**Figure 1**). Myocardial positioning does not itself establish diabetic causality.

**Figure 1:**
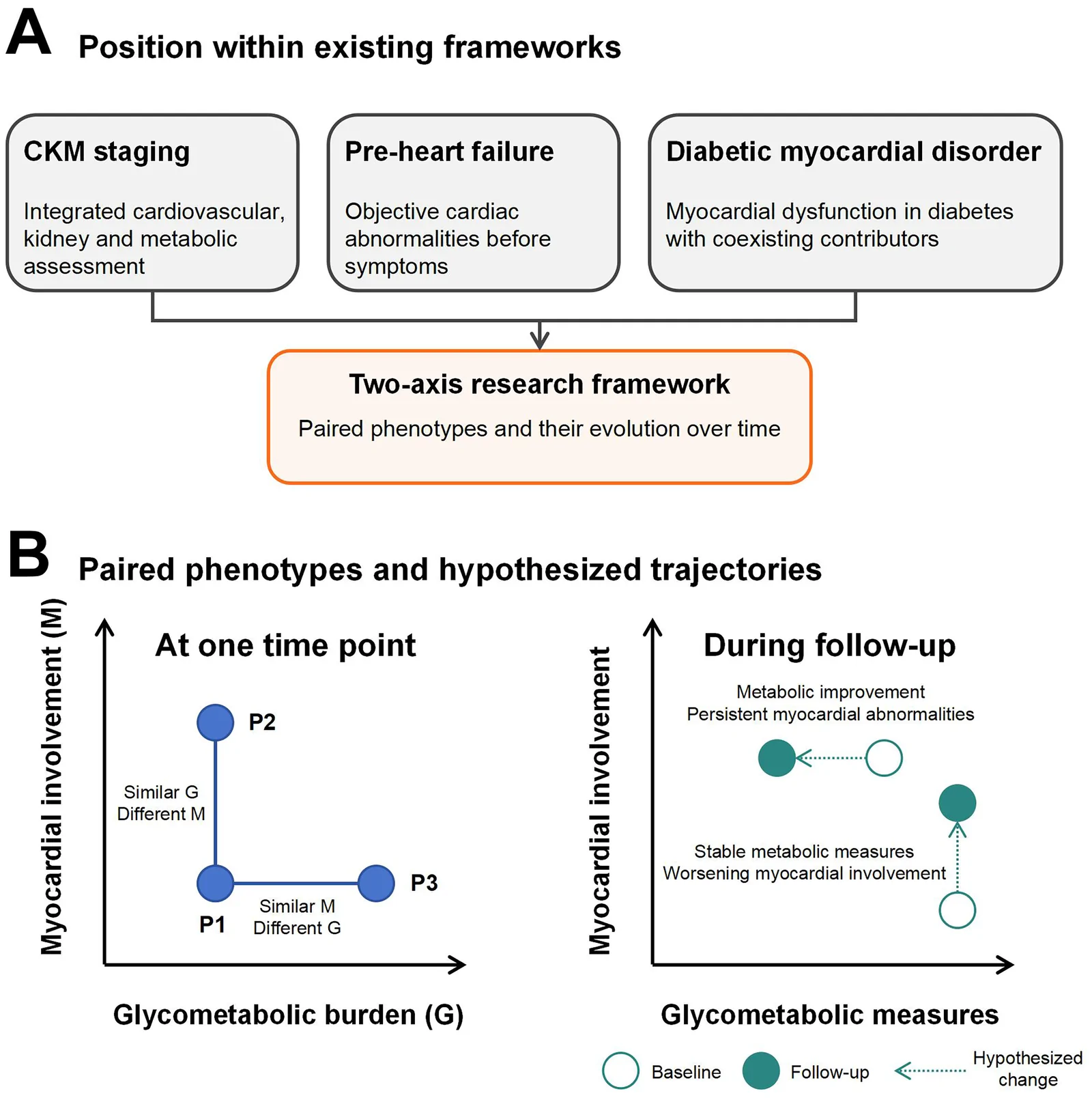
Position within existing frameworks and illustration of the relational two-axis hypothesis. **(A)** Position within existing frameworks. Cardiovascular-kidney-metabolic (CKM) staging integrates cardiovascular, kidney, and metabolic risk and disease. Pre-heart failure identifies objective cardiac abnormalities before symptoms, while diabetic myocardial disorder recognizes heterogeneous myocardial dysfunction in diabetes and the contribution of coexisting conditions. The proposed two-axis hypothesis is positioned within these complementary frameworks and prespecifies the paired metabolic–myocardial trajectory as a longitudinal target of inference rather than proposing a new diagnostic stage or competing clinical classification. **(B)** Paired phenotypes and hypothesized trajectories. At a single time point, individuals may have similar glycometabolic burden (G) but different myocardial involvement (M), or similar myocardial involvement but different glycometabolic burden. P1–P3 represent illustrative individuals. During follow-up, glycometabolic measures may improve while myocardial abnormalities persist, or myocardial involvement may worsen despite apparently stable glycometabolic measures. Open circles indicate baseline and filled circles indicate follow-up. The illustrated positions and trajectories are conceptual and do not represent validated reference values, diagnostic thresholds, or established natural-history sequences. Any empirical classification of relatively greater or lesser myocardial involvement for a given metabolic exposure would require a prespecified conditional reference, must exceed measurement uncertainty, and would require external validation. The illustrated configurations do not establish diabetic causality or biological independence between the two dimensions. CKM, cardiovascular-kidney-metabolic; G, glycometabolic burden; M, myocardial involvement.

The proposal builds on existing frameworks for cardiometabolic and heart failure assessment. Cardiovascular-kidney-metabolic (CKM) staging integrates multiorgan risk and disease, including pre-heart failure, without requiring synchronous metabolic and cardiac progression (13). Pre-heart failure, or Stage B heart failure, identifies asymptomatic structural disease, increased filling pressures, or qualifying biomarker abnormalities (14). Diabetic myocardial disorder recognizes heterogeneous myocardial dysfunction in diabetes and contributions from coexisting conditions (1, 15). The distinction is therefore analytical rather than diagnostic. The framework prespecifies a relational-history hypothesis for testing within, rather than instead of, existing clinical frameworks.

Cross-sectionally, individuals with similar glycometabolic burden but different myocardial involvement, or similar myocardial involvement but different glycometabolic burden, can be compared within and across clinical categories. Such comparisons provide a starting point for investigating cumulative exposure, accompanying conditions, myocardial susceptibility, and measurement factors without establishing causality. They also permit comparisons across the diagnostic threshold for diabetes without treating a change in diagnostic category as evidence of a myocardial transition.

Longitudinally, repeated paired assessment tracks the relationship itself, including changes within an unchanged CKM stage. Glycometabolic measurements might improve while myocardial abnormalities persist, or myocardial involvement might worsen despite apparently stable metabolic measurements. Detailed CKM assessment with biomarkers and imaging can capture the same observations. The proposed contribution is therefore not exclusive analytical capability, but prespecification of the paired trajectory as an object of inference. Whether this improves biological understanding, prediction, or research design remains empirical.

## 4 Biological rationale for metabolic–myocardial heterogeneity

The mechanisms considered here do not validate a distinct two-axis construct. Their narrower role is to provide biological plausibility for why myocardial response may not be an instantaneous or uniform function of current metabolic measurements. Heterogeneity may reflect exposure history, myocardial susceptibility or adaptive capacity, and additional cardiovascular stressors.

Current glycemia does not fully represent the duration or consequences of prior metabolic stress. Ding et al. identified an endothelial CaMK2α/O-GlcNAcylation feedback mechanism that sustained injurious small extracellular vesicle release after glucose normalization in experimental diabetes (16). This finding supports persistence of prior exposure effects, not independence from glycometabolic burden. Apparent discordance between improving glycemic measurements and persistent myocardial abnormalities may therefore reflect metabolic memory. Current glycemia must be distinguished from cumulative exposure when interpreting paired trajectories.

Tissue-specific signaling may also modify the myocardial response to systemic metabolic disturbance. Battiprolu et al. showed that cardiomyocyte-specific FoxO1 deletion protected cardiac structure and function in high-fat-fed mice despite persistent systemic metabolic abnormalities (17). Protection was accompanied by preserved myocardial insulin responsiveness and reduced cardiac lipid accumulation. The experiment supports a modifiable myocardial response but does not establish a naturally occurring protective phenotype in humans.

Additional cardiovascular stressors may amplify or alter the myocardial consequences of metabolic disturbance. Schiattarella et al. induced an experimental heart failure phenotype with preserved ejection fraction through combined metabolic and hypertensive stress in mice (18). Inducible nitric oxide synthase impaired IRE1α/XBP1 signaling, and targeted intervention improved the phenotype. In patients evaluated for suspected coronary disease, Taqueti et al. linked coronary microvascular dysfunction with myocardial injury, diastolic dysfunction, and subsequent heart failure with preserved ejection fraction (19). This observational cohort excluded flow-limiting coronary disease and reduced ejection fraction but did not test two-axis trajectories.

Clinically, worsening GLS despite sustained improvement in HbA1c should not automatically be interpreted as diabetes-specific myocardial progression. Reproducibility of strain measurements, loading conditions, blood pressure, adiposity, kidney function, coronary disease, rhythm, and treatment changes should first be assessed. If these factors do not explain the progression, prior glycemic exposure and delayed tissue effects, including metabolic memory and established myocardial remodeling, become testable contributors rather than assumed causes. The biological proposition is therefore a history- and context-dependent relationship, not independence between metabolic and myocardial disease.

## 5 Operationalization and falsifiable predictions

Neither conceptual axis currently has a validated comprehensive score. One illustrative minimal implementation in people without clinical heart failure pairs time-weighted mean HbA1c over a defined historical window with standardized left ventricular global longitudinal strain (GLS). They are selected for feasibility, continuity, interpretability, and serial assessment, not because they are optimal or comprehensive. The HbA1c mean would equal the area under its time curve divided by the window duration, while GLS could be reported as absolute magnitude. Continuous glucose monitoring metrics, insulin resistance indices, cardiac biomarkers, diastolic parameters, or CMR markers may be preferable for other questions. Measurement choice should follow the biological question.

Diabetes duration, treatment, insulin resistance measures where appropriate, biomarkers, and other imaging findings should be recorded separately rather than automatically combined. Broader composites require justified components and weights, reproducibility testing, and construct validation. Measurements should remain continuous and consistently acquired across follow-up and external datasets. Any empirical classification of relatively greater or lesser myocardial involvement requires a prespecified conditional reference accounting for relevant characteristics and competing cardiac causes, must exceed measurement uncertainty, and requires external validation. Until then, relational descriptors should not be assigned clinically or used to guide treatment.

The central empirical hypothesis is that, after accounting for current and cumulative glycometabolic exposure, treatment, measurement variability, and major competing cardiac causes, preceding paired metabolic–myocardial trajectories retain reproducible information about subsequent myocardial progression beyond contemporaneous measurements alone. This proposition is distinct from simply plotting two dimensions and can be falsified.

For a prespecified population, measurement pair, follow-up interval, comparator model, and minimum meaningful effect, the central hypothesis would be falsified in that setting if, after prespecified accounting for measurement error, cumulative glycometabolic exposure, expected response delay, treatment, and competing cardiac disease, preceding paired trajectories show no reproducible incremental contribution beyond contemporaneous measurements in a conventional model using the same information, with confidence intervals excluding an effect at or above the prespecified minimum meaningful magnitude. An imprecise null result would not suffice. A two-dimensional description could remain, but it would no longer have a demonstrated rationale as an additional explanatory construct.

A practical test could prospectively follow adults with type 2 diabetes without clinical heart failure using serial HbA1c and standardized GLS. An initial 24-month window could define prior glycemic and myocardial trajectories, followed by assessment of subsequent GLS change. A base model would include current HbA1c and GLS, cumulative glycemic exposure, diabetes duration, age, blood pressure, adiposity, kidney function, coronary disease, treatment, loading conditions, and measurement-related factors. A second model would add prespecified prior HbA1c and GLS trajectories and their relationship. Reproducible incremental information from these trajectory terms would support the hypothesis; absence of such information with adequate precision would refute it in that setting. Unexplained residual variation would not be interpreted as diabetes-specific; the relevant glycometabolic signal is the prespecified contribution of measured exposure history and its relation to prior myocardial change. Predictive evaluation should also compare same-information conventional models and an established benchmark such as PREVENT (20).

## 6 Discussion

The rationale for proposing the hypothesis before longitudinal validation is to specify prospectively what should be measured, what relationship should be tested, which alternative explanations must be addressed, and what findings would count against it. Its present purpose is hypothesis generation rather than clinical implementation. Current CKM and heart failure guidance already supports risk-factor management, recognition of pre-heart failure, and evidence-based cardioprotective treatment (13, 14). A two-axis designation does not justify a different drug choice, a new indication, or withdrawal of otherwise indicated therapy.

Potential management applications require separate testing within established prevention pathways. Paired characterization could inform studies of cardiac reassessment timing and intensity among patients receiving guideline-directed prevention. Benefits must exceed current risk-based care while accounting for false-positive findings, additional investigations, and cost. Prevention trials could prespecify both dimensions to examine treatment-response heterogeneity, with phenotype-directed prescribing requiring comparative treatment evidence. Separate metabolic and myocardial response measurements could also identify reproducible patterns for subsequent outcome validation. Neither intensified surveillance nor treatment escalation is currently justified solely by a two-axis profile.

STOP-HF illustrates how an assessment strategy can be tested prospectively (21). Natriuretic peptide screening with collaborative care reduced a composite of ventricular dysfunction and heart failure but did not validate this framework. A two-axis-guided strategy requires comparison with well-implemented CKM and heart failure prevention, using comparable baseline information and retaining all indicated therapies.

The hypothesis should therefore be judged by whether the prespecified relational-history question proves reproducible and informative beyond conventional analyses using the same information. If not, the descriptive diagram alone would not justify an additional explanatory framework. If supported, it could provide a common representation for comparing metabolic–myocardial relationships across time and populations. Biological, prognostic, and clinical applications would still require separate validation.
